# The Moderation Effect of Self-Enhancement on the Group-Reference Effect

**DOI:** 10.3389/fpsyg.2019.01463

**Published:** 2019-06-26

**Authors:** Ruixue Xia, Wanru Su, Fangping Wang, Shifeng Li, Aibao Zhou, Dong Lyu

**Affiliations:** ^1^School of Psychology, Northwest Normal University, Lanzhou, China; ^2^Key Laboratory of Behavioral and Mental Health of Gansu Province, Lanzhou, China; ^3^Department of Social Sciences and Psychology, Baku State University, Baku, Azerbaijan

**Keywords:** group-reference effect, self-enhancement motivation, social identity, ethnic minorities, salience

## Abstract

Previous studies have documented that people tend to respond faster and memorize better to the in-group traits. It may be particularly manifest for ethnic minorities, due to their salient ethnic identity. However, few studies have explored how the valence of traits modulates the in-group preference effect. The present study examined the impacts of ethnic identity salience and the valence of traits on the group-preference effect among 33 Han Chinese in a Tibetan-dominant area and 32 Tibetan participants in a Han-dominant area. Two weeks before the experiment, we measured the ethnic identity salience of participants in both groups. In the formal experiment, we used the group-reference effect (GRE) paradigm with three encoding tasks. The results showed that, regardless of whether ethnic identity was salient, both groups responded faster to positive traits than to negative traits when evaluating their own group, whereas there were no significant difference between the processing of positive traits and negative traits in the out-group evaluation and font judgment tasks. This suggested a pervasive processing advantage of the in-group positive characteristics. The results imply that self-enhancement motivation had a moderation effect on the GRE, as well as the ethnic identity salience may not be necessary for a GRE.

## Introduction

Research on the self can be traced back to the age of ancient Greece. According to Turner et al. (1987), the self-concept includes three basic components: the individual self, the relational self, and the collective self. The collective self reflects the individual’s social identity at the group level. A number of studies have reported that the individual’s social identity may have an impact on how people process information that is related to themselves, e.g., face recognition (Anastasi and Rhodes, 2005; Chiao et al., 2006; Ng et al., 2015; Marsh et al., 2016) and attitudes toward their in-group (Yang et al., 2008; Li H. et al., 2016).

The group-reference effect (GRE) is a kind of processing advantage (e.g., better memory performance, faster reaction time) to the in-group stimuli over the out-group stimuli (Greenwald et al., 1998, 2003; Johnson et al., 2002; Bennett et al., 2010). The in-group is an extension of the self, and people tend to think of themselves as being similar to their in-group (Smith and Henry, 1996). Therefore, individuals may tend to base in-group judgments on the self (Cadinu and Rothbart, 1996).

Ethnic identity is one of the representative identities that an individual can hold. Existing research has shown that ethnic identity can induce the GRE and result in better recognition performance (Mamat et al., 2014). Some studies have also demonstrated that the GRE was more obvious when considered in the context of the salience of the ethnic identity. For example, when Tibetans move to a Han Chinese dominant area, their identity would be highly salient. Therefore, a significant in-group (ethnic) memory advantage was observed; however, no such effect was observed for the Han Chinese, who were already dominant in the area (Yang et al., 2008). A study with Tibetan students who lived in a predominantly Tibetan area showed that when ethnic identity was primed, a significant in-group effect on memory performance was present (Li H. et al., 2016). These findings demonstrated that ethnic identity could induce the GRE, and it might be modulated by the salience of the individual’s ethnic identity. Therefore, the first aim of the present study was to replicate the modulation effect of the salience of ethnic identity on GRE in Tibetan and Han Chinese who lived where their ethnicity was not dominant.

Based on self-enhancement viewpoints, individuals tend to think that they and their in-group have better personality traits than others and out-groups (Sedikides and Gregg, 2008; Alicke and Sedikides, 2009; Sedikides and Alicke, 2012) while sorting the combination of self/other and positive/negative traits (Brown, 1986; Dunning et al., 1989; Gebauer et al., 2012). Thus, positive traits would be integrated quickly and automatically into the self-concept or the in-group concept. Meanwhile, negative traits would be excluded from the self-concept or the in-group concept (Watson et al., 2007; Cai et al., 2016). This self-enhancement effect can be observed even without any explicit requirement for self-referential appraisal (Herbert et al., 2011). Therefore, people may process positive traits and negative traits differently in the GRE paradigm. However, most studies on GRE did not differentiate the valances of traits. The second aim of the present study was to explore how self-enhancement influences the GRE and examine the interaction between self-enhancement and the salience of ethnic identity.

In the present study, we examined the effect of ethnic identity salience and the valence of the traits on GRE in two groups of participants. One group was Han Chinese, which came from a Tibetan dominant area, the other group was Tibetan, which came from a Han Chinese dominant area. Most studies have used memory performance as the GRE index (Johnson et al., 2002; Yang and Huang, 2007; Bennett et al., 2010; Liu et al., 2015; Lee et al., 2016). However, in the present study we used reaction times (RT) as the GRE index. RT have been well documented as sensitive in differentiating between the in-group’s and out-group’s attitudes and cognitive processing (Greenwald et al., 2003; Cai et al., 2016). We expected that the GRE effect would primarily occur in positive trait judgments and would be modulated by self-enhancement.

## Materials and Methods

### Participants

Thirty-two Tibetan students (17 female and 15 male, Mean age = 20.25 years, *SD* = 1.34) were recruited from Northwest Normal University and thirty-three Han Chinese students (23 female and 10 male, mean age = 22.56 years, *SD* = 0.77) were recruited from Gansu Normal University for Nationalities. All participants had normal or corrected-to-normal vision, and no participants had any prior experience with this study. All students were paid for their participation. The scientific and research Ethics Committee of the School of Psychology, Northwest Normal University approved the experimental protocol, and written informed consent was obtained from all participants prior to the study. The participants were free to withdraw at any point in the experiment without penalty. If, for any reason, they did not feel comfortable during this study, they could leave the laboratory. Participants were informed that all information they provided would remain confidential and would not be associated with their name. The experiment lasted for approximately 30 min, and participants received feedback regarding their results after they completed the tasks.

### Experimental Design

A 3 (encoding task: Han Chinese vs. Tibetan vs. font) × 2 (valence: positive vs. negative) × 2 (ethnic group: Tibetan vs. Han Chinese) mixed experimental design was conducted, with both encoding task and valence as within-subjects variables, and ethnic group as a between-subjects variable. Reaction time was the dependent variable.

### Stimulus Material

The stimuli of the current study included 126 (half positive and half negative) personality trait adjectives. The participants first practiced the task with six words (three positive and three negative). The remained 120 words were used in the formal experimental task. The adjectives were selected from existing studies (Zhou et al., 2013; Li S. et al., 2016). All selected words were two-character trait adjectives. We matched the familiarity (*M*_pos._ = 5.69, *SD* = 0.94, *M*_neg._ = 5.68, *SD* = 1.23), frequency (*M*_pos._ = 22.52 per million, *M*_neg._ = 20.63 per million) and the number of strokes (*M*_pos._ = 16.52, *SD* = 3.73, *M*_neg._ = 17.30, *SD* = 4.51) of the words. *T*-test showed no significant differences between the positive and negative words on the three dimensions (*p*s > 0.05). Moreover, the valence of words (*M*_pos._ = 5.12, *SD* = 0.64, *M*_neg._ = 2.24, *SD* = 0.62) differed significantly between the positive and negative words (*p* < 0.05). In order to avoid mutual interference between the various encoding tasks, each word was presented only once across all judgment tasks. Each word was randomly assigned to one encoding tasks for each participant. 63 positive and 63 negative trait adjectives were evenly distributed across the three encoding tasks.

### Experimental Tasks

Participants were asked to perform three encoding tasks, respectively, the in-group encoding task, the out-group encoding task, and the font structure judgment task. In the in-group and the out-group encoding tasks, the participants were asked to make judgments on whether the trait words presented on the screen appropriately described people of their own or another ethnic group. For the Tibetan participants, judgments of Han Chinese was the out-group encoding task; for the Han Chinese, judgments of the Tibetans was the out-group encoding task. As a control condition, the participants were asked to judge the words’ font structure (Are there characters with left-right structure?). Participants responded to each of the stimuli by pressing the appropriate keys. The keys for each of the participants were counterbalanced. Half of the participants were instructed to press “f” for “yes” and “j” for “no,” while the other half were given the opposite instructions. All stimuli were randomly presented.

### Procedure

Two weeks before the experiment all participants were asked to complete the Twenty Statements Test (Kuhn and Mcpartland, 1954). This test is often used to examine whether one’s ethnic identity is salient in their self-schema. The more salient the ethnic identity, the more likely it is to be mentioned (Yang et al., 2008).

Before the formal experimental task, each of the participants performed a six-trial (two trials for each encoding task) practice. The six trials were identical to the formal experimental tasks. The participants’ reaction time to the practice trials were not included in the data analysis. Each trial started with a fixation cross at the center of the screen for 500 ms and a follow-up blank screen for 250 ms. Next, a trait adjective was presented together with a label for the current encoding tasks (Han Chinese, Tibetan, or font) above it until the subject made a response. Three encoding tasks were randomly distributed in each of the trials. Then a blank screen showed up for 500 ms before the next trial. The stimuli procedure were programed and presented using E-Prime 2.0. The data were analyzed using the SPSS 21.0.

## Results

### Ethnic Identity Salience

Tibetan participants showed higher ethnic identity salience than Han participants. In the Twenty Statements Test (Kuhn and Mcpartland, 1954), 29 of the 32 Tibetan participants mentioned their own ethnic identity, such as “I am a Tibetan” or “I am from Tibet.” However, only 5 of 33 Han participants mentioned their own ethnic identity, χ^2^ (1, *N* = 65) = 37.10, *p* < 0.001.

### Reaction Times

Responses were scored if the appropriate key was pressed between 300 and 3000 ms after the adjective was displayed on the screen. A mixed measures ANOVA was conducted with encoding task and valence as within-subject variables and ethnic group as a between-subjects variable. The results showed that the main effects of encoding task, ethnic group, and valence were not significant *F*s < 2.94, *p*s > 0.05. Also, the interactions between encoding task and valence, and ethnic group and valence were also not significant, *F*s < 2.13, *ps* > 0.05.

**FIGURE 1:**
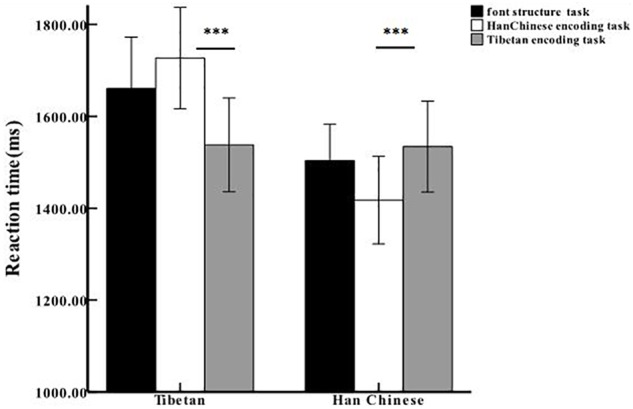
Reaction time in three encoding tasks for Tibetan and Han Chinese. ^∗∗∗^*p* < 0.001. Error bars represent one standard error from the mean.

However, there was a significant interaction between encoding task and ethnic group, *F*_(2,63)_ = 13.59, *p* < 0.001, $\eta_{p}^{2}$ = 0.18. A simple effects analysis showed that the trait judgment for the in-group was significantly faster than that for out-group (Bonferroni-corrected *p*s < 0.001, Cohen’s *d*s ≥ 1.67) for both Tibetans and Han Chinese, and Tibetans also responded faster for the in-group encoding task than for the font encoding task (Bonferroni-corrected *p* < 0.01, Cohen’s *d* = 1.80).Whereas there was no significant difference between the out-group and the font encoding tasks (*p*s ≥ 0.13) (shown in Table 1 and Figure 1). These results suggest that both Tibetan and Han Chinese exhibited the advantage effect of the in-group reference processing.

**Table 1:** The mean scores and standard deviance of Reaction time (ms) in three encoding tasks for Tibetan and Han Chinese.

	Tibetan encoding task		Han Chinese encoding task		Font structure task	
	M	SD	M	SD	M	SD
Tibetan	1537.79	69.95	1726.67	71.46	1661.14	66.75
Han Chinese	1534.29	68.88	1417.78	70.37	1503.82	65.73


**FIGURE 2:**
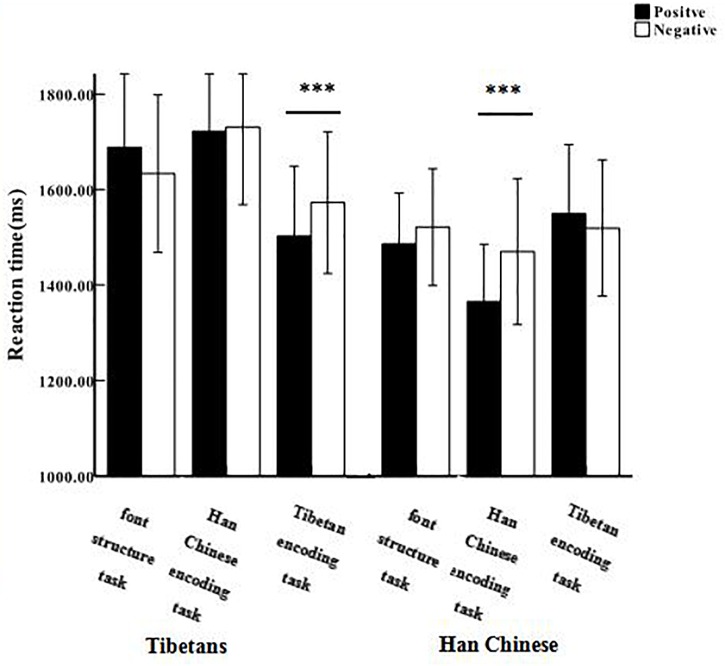
Reaction time of positive and negative adjective in three encoding tasks for Tibetan and Han Chinese. ^∗∗∗^*p* < 0.001. Error bars represent one standard error from the mean.

Importantly, the results also revealed a significant three-way interaction for encoding task, valence, and ethnic group, *F*_(2,63)_ = 5.96, *p* < 0.01, η^2^*_p_* = 0.09. A simple effects analysis comparing valences indicated that for both Tibetans and Han Chinese, participants showed faster RT for the in-group positive traits than that for negative traits (*p*s < 0.05, Cohen’s *d*s ≥ 0.97). However, there was no significant difference for trait valence both in the out-group (*p*s > 0.05) and the font encoding tasks (*p*s > 0.05). These results suggested that the processing superiority effect on the in-group positive traits was apparent for both Tibetans and Han Chinese (shown in Table 2 and Figure 2).

**Table 2:** The mean scores and standard deviance of Reaction time (ms) of positive and negative adjective in three encoding tasks for Tibetan and Han Chinese.

	Tibetan encoding task						Han Chinese encoding task						Font structure task			
	Positive		Negative		*P*	*d*	Positive		Negative		*P*	*d*	Positive		Negative		*P*	*d*
	M	SD	M	SD			M	SD	M	SD			M	SD	M	SD		
Tibetan	1502.88	71.94	1572.71	72.06	0.045	–0.97	1722.46	69.49	1730.88	77.87	0.082	–0.11	1688.78	66.04	1633.49	71.50	0.104	0.80
Han Chinese	1549.33	70.85	1519.24	70.95	0.374	0.42	1365.43	68.43	1470.12	76.68	0.005	–1.44	1486.24	65.03	1521.39	70.41	0.291	–0.52


Another simple effects analysis of comparing encoding tasks also showed that for both Tibetans and Han Chinese, judgments for the in-group positive characteristics were significantly faster than that for the out-group characteristics (Bonferroni-corrected *p*s < 0.001, Cohen’s *d*s ≥ 2.64) and the font encoding task (Bonferroni-corrected *p*s ≤ 0.01, Cohen’s *d*s ≥ 1.81), and no significant differences between the font and out-group encoding tasks were observed (*p*s ≥ 0.24). For the negative characteristics, Han Chinese showed no significant difference among three encoding tasks for in-group, out-group and font (*p*s ≥ 0.26), however, Tibetan participants showed faster RT in the in-group encoding tasks than in the out-group tasks (Bonferroni-corrected *p* = 0.001, Cohen’s *d* = 2.11).

Finally, due to the significant difference of salience of ethnic identity that was found between Tibetan and Han Chinese, we repeated the analyses of reaction time with ethnic identity salience as a covariate variable and similar results were obtained with and without ethnic identity salience in the analyses.

## Discussion

The current study explored how self-enhancement motivation and the salience of ethnic identity affected GRE among Tibetan and Han Chinese groups at two universities. Firstly, the results indicated that Tibetan participants had stronger ethnic identity salience than Han Chinese participants, even though Tibetan participants were in a Han Chinese dominant area and the Han Chinese participants were in a Tibetan dominant area. Both groups demonstrated significantly faster RT under the in-group encoding task in comparison to the out-group encoding and font encoding tasks. This was the case regardless of ethnic identity salience and confirmed the existence of the GRE. Most importantly, both Tibetans and Han Chinese exhibited faster RT when judging positive traits, rather than negative traits, in the in-group encoding task. However, in the out-group and the font encoding tasks, no such valence effect was observed. These findings suggested that self-enhancement motivation modulates the GRE.

In the present study, Tibetan participants exhibited higher ethnic identity salience than did the Han Chinese students. These results are consistent with previous findings on ethnic identity for Tibetan groups (Yang et al., 2008; Li H. et al., 2016). The present study’s Tibetan participants came from a predominantly Han Chinese university and the Han Chinese participants came from a predominantly Tibetan university. However, Han Chinese is considered the default ethnic category in China, due to being the majority ethnicity, and is seldom reflected upon by its members (Yang et al., 2008), and ethnic identity may not be as salient in the Han Chinese self-concept in comparison to the Tibetan self-concept (Mamat et al., 2014; Li H. et al., 2016). Since Tibetan is an ethnic minority group in China, Tibetan students living in a Han Chinese dominant area may frequently confront issues surrounding their ethnic identity. Thus, ethnic identity may be a salient characteristic of their overall self-concept (Li H. et al., 2016). Illustrating this salience in other groups, Mcguire et al. (1978) demonstrated that Black ethnic minorities in the United States of America spontaneously mentioned their ethnicity when asked to answer the question “Who am I?” Therefore, ethnic identity being a salient characteristic within the self-concept of minority groups is an over-arching phenomenon and not specific to the Tibetan population.

Regardless of the salience of their ethnic identity, both Tibetans and Han Chinese showed significant GRE in the present study. These results are congruent with previous studies on GRE (Johnson et al., 2002; Bennett et al., 2010). For example, Bennett et al. (2010) reported that in-group referential recall was significantly greater than out-group referential recall. The collective identities (e.g., ethnic) provide a useful organizational framework, which would facilitate memory due to the organizational, elaborative, mental cueing, or evaluative properties of the group-reference encoding task, or the combination of these characteristics (Johnson et al., 2002). The present study further showed that such organizational framework would also facilitate the categorization of the in-group information. However, our results were inconsistent with those of Yang et al. (2008) and Li H. et al. (2016), which demonstrated that Han Chinese participants did not show a significant GRE. One possible explanation for this inconsistency may be the use of a different index in the present and previous studies (Johnson et al., 2002; Bennett et al., 2010; Liu et al., 2015; Lee et al., 2016). In contrast to using memory performance, as done by Yang et al. (2008) and Li H. et al. (2016), RT is a more sensitive index for categorizing and measuring inter-group attitudes (Greenwald et al., 2003; Watson et al., 2007; Gebauer et al., 2012). The present study used RT rather than memory performance and found that Han Chinese participants also showed a significant GRE. This suggested that extrinsic ethnic identity salience might not be a necessary condition for inducing the GRE. It should be noted that the group descriptiveness task itself may highlight ethnic identity and make in-group membership very salient and relevant on a situational level. This may also have contributed to the GRE observed for the two groups in the present study. Further studies are needed to examine whether the GRE depends on which index is used. Also, future studies should use an implicit task to examine how ethnic identity salience affects the GRE.

The present results showed that GRE was modulated by the self-enhancement motivation. Both Tibetans and Han Chinese showed faster judgments for the in-group positive traits than that for negative traits. However, this tendency toward positive response priority was not found in the out-group encoding task or the font encoding task. These results suggested that individuals tend to integrate positive traits more easily than negative traits into their in-group members (Dasgupta, 2004). This tendency of more easily recognizing positive personality characteristics of one’s own ethnic group may satisfy their needs for self-enhancement and self-esteem (Sedikides et al., 2013). The pursuit of self-enhancement, which is prevalent across persons, groups, nations, and cultures, promotes and helps to maintain a positive self-schema (Hepper et al., 2013). Such self-enhancement motivation would lead to the individuals’ evaluating their own membership group as more favorable (e.g., inter-group bias), and, sometimes, even to derogate the out-group (e.g., group-serving bias). In-group preference is an essential human characteristic, and in-group favoritism is a form of self-identification (Tajfel and Turner, 1986). People tend to evaluate in-group members more positively, thereby validate their own cultural worldview (Hewstone et al., 2002; Sedikides et al., 2013). Therefore, in-group preference plays an important role in individual survival and social adaptation. An interesting outcome of the present study was that the Tibetan participants responded faster to the negative traits in the in-group encoding task than to those in the out-group or the font encoding tasks. A possible explanation may be that the faster responses to negative traits by Tibetan participant means that they tend to avoid the in-group negative information as quickly as possible. This may further confirm the self-enhancement/self-protection effect.

## Conclusion

In summary, the present study expanded the findings of the previous research by demonstrating that self-enhancement motivation moderates the GRE. That is, ethnic identity salience may not be necessary for GRE when self-enhancement is considered. Further research should verify the effect of self-enhancement motivation on GRE in different paradigms (explicit vs. implicit) and different contexts (dominant groups vs. non-dominant groups), and examine the different indicators (reaction time vs. memory performance) of GRE.

## Data Availability

All datasets generated for this study are included in the manuscript and/or the Supplementary Files.

## Ethics Statement

The School of Psychology, Northwest Normal University, 967 East Anning Road, Lanzhou, 730070, China. Tel.: +86-0931-7975316.

PROTOCOL

(1) Introduction

Previous studies have documented that people tend to respond faster and memorize in-group traits better. This process of superiority is called the group-reference effect (GRE). It may be particularly manifest for ethnic minorities, due to their salient ethnic identity. However, few studies have explored how the valence of traits may modulate this in-group preference effect. According to self-enhancement motivation theory, individuals may only show preference for positive traits rather than negative traits.

(2) Ethical issues

1. In this study we will ask participants to watch the picture on computer screen and make a button response.
2. If participants agree to participate, they are free to withdraw at any time throughout the duration of the experiment without any penalty.
3. During the study if the participants feel uncomfortable, they may leave the laboratory and participants’ information will be discarded.
4. All information participants provide will remain confidential and will not be associated with participants’ name.
5. This study will last for approximately 30 min.
6. Participants were informed of their rights and were free to ask any questions concerning the research.
7. If participants can finish all the experiments, he or she may obtain financial compensation.
8. If participants have any further questions concerning this study please feel free to contact us.

(3) Objectives

We expected that the GRE effect would primarily occur in positive trait judgment and would be motivated by self-enhancement.

(4) Enrollment Procedures

By posting ads on campus, recruit the participant.

(5) What is collected?

(5.1) Behavioral results:

According to the button responses of subjects, record Reaction Times.

Contact:

Researcher: Wanru Su

Email address: 2514439660@qq.com

Tel.: +86-15101210805

Supervisor: AiBao Zhou

Email address: zhouab@nwnu.edu.

Tel.: +86-0931-7975075

The scientific and research Ethics Committee of the School of Psychology, NWNU.

## Author Contributions

AZ conceived the research. RX participated in writing the manuscript. WS participated in performing the research. FW participated in reviewing the literatures. DL participated in making the figures and tables. SL participated in modifying the manuscript.

## Conflict of Interest Statement

The authors declare that the research was conducted in the absence of any commercial or financial relationships that could be construed as a potential conflict of interest.

## References

[B1] Alicke M. D. Sedikides C. (2009). Self-enhancement and self-protection: what they are and what they do. *Eur. Rev. Soc. Psychol.* 20 1–48. 10.1080/10463280802613866

[B2] Anastasi J. S. Rhodes M. G. (2005). An own-age bias in face recognition for children and older adults. *Psychon. Bull. Rev.* 12 1043–1047. 10.3758/bf03206441 16615326

[B3] Bennett M. Allan S. Anderson J. Asker N. (2010). On the robustness of the group reference effect. *Eur. J. Soc. Psychol.* 40 349–354. 10.1002/ejsp.630

[B4] Brown J. D. (1986). Evaluations of self and others: self-enhancement biases in social judgments. *Soc. Cogn.* 4 353–376. 10.1521/soco.1986.4.4.353 19487485

[B5] Cadinu M. R. Rothbart M. (1996). Self-anchoring and differentiation processes in the minimal group setting. *J. Personal. Soc. Psychol.* 70 661–677. 10.1037/0022-3514.70.4.661 8636892

[B6] Cai H. Wu L. Shi Y. Gu R. Sedikides C. (2016). Self-enhancement among Westerners and Easterners: a cultural neuroscience approach. *Soc. Cogn. Affect. Neurosci.* 11 1569–1578. 10.1093/scan/nsw072 27217110 PMC5040913

[B7] Chiao J. Y. Heck H. E. Nakayama K. Ambady N. (2006). Priming race in biracial observers affects visual search for Black and White faces. *Psychol. Sci.* 17 387–392. 10.1111/j.1467-9280.2006.01717.x 16683925

[B8] Dasgupta N. (2004). Implicit ingroup favoritism, outgroup favoritism, and their behavioral manifestations. *Soc. Justice Res.* 17 143–169. 10.1023/b:sore.0000027407.70241.15

[B9] Dunning D. Meyerowitz J. A. Holzberg A. D. (1989). Ambiguity and self-evaluation: the role of idiosyncratic trait definitions in self-serving assessments of ability. *J. Personal. Soc. Psychol.* 57 1082–1090. 10.1037/0022-3514.57.6.1082

[B10] Gebauer J. E. Göritz A. S. Wilhelm H. Constantine S. (2012). Self-Love or other-love? Explicit other-preference but implicit self-preference. *PLoS One* 7:e41789. 10.1371/journal.pone.0041789 22848605 PMC3405013

[B11] Greenwald A. G. McGhee D. E. Schwartz J. L. K. (1998). Measuring individual differences in implicit cognition: the implicit association test. *J. Personal. Soc. Psychol.* 74 1464–1480. 10.1037/0022-3514.74.6.1464 9654756

[B12] Greenwald A. G. Nosek B. A. Banaji M. R. (2003). Understanding and using the implicit association test: I. An improved scoring algorithm. *J. Personal. Soc. Psychol.* 85 197–216. 10.1037/0022-3514.85.2.197 12916565

[B13] Hepper E. G. Sedikides C. Cai H. (2013). Self-Enhancement and self-protection strategies in China: cultural expressions of a fundamental human motive. *J. Cross Cult. Psychol.* 44 5–23. 10.1177/0022022111428515

[B14] Herbert C. Pauli P. Herbert B. M. (2011). Self-reference modulates the processing of emotional stimuli in the absence of explicit self-referential appraisal instructions. *Soc. Cogn. Affect. Neurosci.* 6 653–661. 10.1093/scan/nsq082 20855295 PMC3190208

[B15] Hewstone M. Rubin M. Willis H. (2002). Intergroup bias. *Annu. Rev. Psychol.* 53 575–604. 10.1146/annurev.psych.53.100901.135109 11752497

[B16] Johnson C. Gadon O. Carlson D. Southwick S. Faith M. Chalfin J. (2002). Self-reference and group membership: evidence for a group-reference effect. *Eur. J. Soc. Psychol.* 32 261–274. 10.1002/ejsp.83

[B17] Kuhn M. H. Mcpartland T. S. (1954). An empirical investigation of self-attitudes. *Am. Sociol. Rev.* 19 68–76. 10.2307/2088175 9871372

[B18] Lee H. N. Rosa N. M. Gutchess A. H. (2016). Ageing and the group-reference effect in memory. *Memory* 24 746–756. 10.1080/09658211.2015.1049184 26252870

[B19] Li H. Wang E. X. Jin S. Wu S. (2016). Ethnic identity salience improves recognition memory in tibetan students via priming. *Cult. Divers. Ethn. Minor. Psychol.* 22 229–236. 10.1037/cdp0000051 26147632

[B20] Li S. Xu K. Xu Q. Xia R. Ren D. Zhou A. (2016). Positive bias in self-appraisals from friend’s perspective: an event-related potential study. *Neuroreport* 27 694–698. 10.1097/WNR.0000000000000599 27138953

[B21] Liu Z. Wu L. Hou C. (2015). Social identity: the cause of distinction between group-reference and self-reference effects. *Soc. Behav. Personal. Int. J.* 43 1409–1418. 10.2224/sbp.2015.43.9.1409

[B22] Mamat M. Huang W. Shang R. Zhang T. Li H. Wang Y. (2014). Relational self versus collective self: a cross-cultural study in interdependent self-construal between Han and Uyghur in China. *J. Cross Cult. Psychol.* 45 959–970. 10.1177/0022022114530558

[B23] Marsh B. U. Pezdek K. Ozery D. H. (2016). The cross-race effect in face recognition memory by bicultural individuals. *Acta Psychol.* 169 38–44. 10.1016/j.actpsy.2016.05.003 27219532

[B24] Mcguire W. J. Mcguire C. V. Child P. Fujioka T. (1978). Salience of ethnicity in the spontaneous self-concept as a function of one’s ethnic distinctiveness in the social environment. *J. Personal. Soc. Psychol.* 36 511–520. 10.1037/0022-3514.36.5.511 671213

[B25] Ng A. H. Steele J. R. Sasaki J. Y. Sakamoto Y. Williams A. (2015). Culture moderates the relationship between interdependence and face recognition. *Front. Psychol.* 6:1620. 10.3389/fpsyg.2015.01620 26579011 PMC4621394

[B26] Sedikides C. Alicke M. D. (2012). “Self-enhancement and self-protection motives,” in *Oxford handbook of Motivation*, ed. Ryan R. M. (New York, NY: Oxford University Press), 10.1093/oxfordhb/9780195399820.013.0017

[B27] Sedikides C. Gaertner L. Luke M. A. O’Mara E. M. Gebauer J. (2013). A three-tier hierarchy of motivational self-potency: individual self, relational self, collective self. *Adv. Exp. Soc. Psychol.* 48 235–296. 10.1016/B978-0-12-407188-9.00005-3 27378977

[B28] Sedikides C. Gregg A. P. (2008). Self-enhancement: food for thought. *Perspect. Psychol. Sci.* 3 102–116. 10.1111/j.1745-6916.2008.00068.x 26158877

[B29] Smith E. Henry S. (1996). An in-group becomes part of the self: response time evidence. *Personal. Soc. Psychol. Bull.* 22 635–642. 10.1177/0146167296226008

[B30] Tajfel H. Turner J. C. (1986). The social identity theory of intergroup behavior. *Psychol. Intergroup Relat.* 13 7–24.

[B31] Turner J. C. Hogg M. A. Oakes P. J. Reicher S. D. Wetherell M. S. (1987). Rediscovering the social group: a self-categorization theory. *Br. J. Soc. Psychol.* 26 347–348. 10.1111/j.2044-8309.1987.tb00799.x 22390752

[B32] Watson L. A. Dritschel B. Obonsawin M. C. Jentzsch I. (2007). Seeing yourself in a positive light: brain correlates of the self-positivity bias. *Brain Res.* 1152 106–110. 10.1016/j.brainres.2007.03.049 17462610

[B33] Yang H. Huang X. (2007). Group-reference effect in chinese. *Acta Psychol. Sin.* 39 235–241.

[B34] Yang H. Liao Q. Huang X. (2008). Minorities remember more: the effect of social identity salience on group-referent memory. *Memory* 16 910–917. 10.1080/09658210802360629 18785055

[B35] Zhou A. Li S. Herbert C. Xia R. Xu K. Xu Q. (2013). Perspective taking modulates positivity bias in self-appraisals: behavioral and event-related potential evidence. *Soc. Neurosci.* 8 326–333. 10.1080/17470919.2013.807873 23802122

